# Evaluation of the characteristics of infection prevention and control programs and infection control committees in Brazilian hospitals: A countrywide cross-sectional study – CORRIGENDUM

**DOI:** 10.1017/ash.2023.189

**Published:** 2023-06-19

**Authors:** Beatriz Arns, Crepin Aziz Jose Oluwafoumi Agani, Guilhermo Prates Sesin, Jaqueline Driemeyer C. Horvath, Débora Vacaro Fogazzi, Fernanda Kelly Romeiro Silva, Lauren Sezera Costa, Adriano Jose Pereira, Antônio Paulo Nassar Junior, Bruno Tomazini Cavalcanti, Camila Dietrich, Viviane Cordeiro Veiga, Daniela G.M. Catarino, Maysa Yukari Cheno, Alexandre Biasi, Bianca Ramos Ferronatto, Bil Randerson Bassetti, Caio Cesar Ferreira Fernandes, Caroline Deutschendorf, Cintia Magalhães Carvalho Grion, Claudia Fernanda de Lacerda Vidal, Cláudio Dornas de Oliveira, Eliana Bernadete Caser, Emerson Boschi, Everton Macêdo Silva, Felipe Dal Pizzol, Hugo Correa de Andrade Urbano, Iany Silva, Israel Silva Maia, Leila Rezegue de Moraes Rego, Luana Pontes Oliveira, Maria Brandão Tavares, Marianna Deway Andrade Dracoulakis, Marina Peres Bainy, Nicole Alberti Golin, Pablo Oscar Tomba, Pedro Martins Pereira Kurtz, Rafael Botelho Foernges, Rejane Martins Prestes, Rodrigo Morel Vieira de Melo, Rodrigo Reghini Da Silva, Tatiana Gozzi Pancev Toledo, Valéria Paes Lima, Vanildes de Fátima Fernandes, Wilson José Lovato, Alexandre Prehn Zavascki

In the above article^1^, author names Bruno Tomazini and Alexandre Biasi Cavalcanti were erroneously spelled; this has since been corrected.
